# Supplementary material for: Quality of first antenatal care visits and perinatal outcomes: Evidence from a cohort study in Ethiopia, Kenya, South Africa, and India
Source: PLOS Glob Public Health. 2026 Apr 13;6(4):e0006248. doi: 10.1371/journal.pgph.0006248 (PMC13075702; doi:10.1371/journal.pgph.0006248)
Supplement: S1 Appendix — Description of regression models. Table B. Proportion of pregnant women who received each of the six and all six care components at first ANC visits in four countries by research sites. Table C. Proportion of pregnant women who received each of the six and all six care components at first ANC visits in four countries by facility ownership. Table D. Results of mixed-effect logistic regressions (ANC quality as a continuous score). (DOCX) [file pgph.0006248.s001.docx]

**Quality of first antenatal care visits and perinatal outcomes: evidence from a cohort study in Ethiopia, Kenya, South Africa, and India**

**Supplemental File 1**

**Table A. Description of regression models**

| **Models** | | | **Sample** | **Independent variable** | **Outcome** |
| --- | --- | --- | --- | --- | --- |
| Primary analysis | | 1. Primary analysis assessed the association between good ANC quality at the first visit (6 care components) and fetal losses | Pregnant women who were enrolled in the first trimester | 6 care components at the first visit | fetal losses |
|  |  | 2. Primary analysis assessed the association between good ANC quality at the first visit (5 care components) and fetal losses | Pregnant women who were enrolled in the first trimester | 5 care components at the first visit | fetal losses |
|  |  | 3. Primary analysis assessed the association between good ANC quality at the first visit (6 care components) and LBW newborns | Pregnant women who were enrolled in any trimester | 6 care components at the first visit | LBW newborns |
|  |  | 4. Primary analysis assessed the association between good ANC quality at the first visit (5 care components) and LBW newborns | Pregnant women who were enrolled in any trimester | 5 care components at the first visit | LBW newborns |
| Sensitivity analysis | Fetal regressions,  restricted to those with more reliable GA  dining | 5. Sensitivity analysis assessed the association between good ANC quality at the first visit (6 care components) and fetal losses | Pregnant women who were enrolled in the first trimester and had more reliable GA dating | 6 care components at the first visit | fetal losses |
|  |  | 6. Sensitivity analysis assessed the association between good ANC quality at the first visit (5 care components) and fetal losses. | Pregnant women who were enrolled in the first trimester and had more reliable GA dating | 5 care components at the first visit | fetal losses |
|  | LBW regressions,  restricted to those with actual newborn  birthweight available | 7. Sensitivity analysis assessed the association between good ANC quality at the first visit (6 care components) and LBW newborns | Pregnant women who were enrolled in any trimester and those with babies’ actual birthweight data available | 6 care components at the first visit | LBW newborns |
|  |  | 8. Sensitivity analysis assessed the association between good ANC quality at the first visit (5 care components) and LBW newborns | Pregnant women who were enrolled in any trimester and those with babies’ actual birthweight data available | 5 care components at the first visit | LBW newborns |
|  | Models 1, 3, 5, and 7 with the independent variable as continuous ANC score | 9. Sensitivity analysis assessed the association between good ANC quality (continuous score) and fetal losses | Pregnant women who were enrolled in the first trimester | Continuous score (0 to 100%) | fetal losses |
|  |  | 10. Sensitivity analysis assessed the association between good ANC quality at the first visit (continuous score) and LBW newborns | Pregnant women who were enrolled in any trimester | Continuous score (0 to 100%) | LBW newborns |
|  |  | 11. Sensitivity analysis assessed the association between good ANC quality at the first visit (continuous score) and fetal losses | Pregnant women who were enrolled in the first trimester and had more reliable GA dating | Continuous score (0 to 100%) | fetal losses |
|  |  | 12. Sensitivity analysis assessed the association between good ANC quality at the first visit (continuous score) and LBW newborns | Pregnant women who were enrolled in any trimester and those with babies’ actual birthweight data available | Continuous score (0 to 100%) | LBW newborns |

**Table B.** **Proportion of pregnant women who received each of the six and all care components at first ANC visit in four countries by research site**

| **ANC quality care components** | **Ethiopia**  **N=883** | | **Kenya**  **N=896** | | **South Africa**  **N=854** | | **India**  **N=967** | |
| --- | --- | --- | --- | --- | --- | --- | --- | --- |
|  | **East Shewa**  **(rural)  N=451** | **Adama Town**  **(urban) N=432** | **Kitui**  **(rural) N=459** | **Kiambu**  **(urban)  N=437** | **Nongoma**  **(rural)  N=436** | **uMhlathuze**  **(urban)  N=418** | **Sonipat**^3^**N=489** | **Jodhpur**^3^**N=478** |
|  | **Frequency (%)** | **Frequency (%)** | **Frequency (%)** | **Frequency(%)** | **Frequency (%)** | **Frequency (%)** | **Frequency (%)** | **Frequency (%)** |
| **Physical examination** |  |  |  |  |  |  |  |  |
| Blood pressure measurement | 252 (55.9%) | 388 (89.8%) | 432 (94.1%) | 429 (98.4%) | 434 (99.5%) | 418 (100%) | 489 (100%) | 465 (97.5%) |
| **Test and examination** |  |  |  |  |  |  |  |  |
| Blood test (blood draw or finger prick) | 411 (91.1%) | 427 (98.8%) | 446 (97.2%) | 430 (98.6%) | 436 (100%) | 417 (99.8%) | 421 (86.1%) | 434 (91.0%) |
| Urine test | 325 (72.1%) | 427 (98.8%) | 399 (86.9%) | 402 (92.2%) | 434 (99.5%) | 416 (99.5%) | 358 (73.2%) | 287 (60.0%) |
| Ultrasound | 140 (31.1%) | 243 (56.3%) | 18 (3.9%) | 56 (12.9%) | 2 (0.5%) | 63 (15.1%) | 59 (12.1%) | 128 (27.1%) |
| **Counseling** |  |  |  |  |  |  |  |  |
| Signs of pregnancy complications | 102 (22.6%) | 171 (39.9%) | 218 (47.6%) | 326 (74.8%) | 220 (50.7%) | 200 (48.0%) | 22 (4.5%) | 121 (25.6%) |
| **Preventions** |  |  |  |  |  |  |  |  |
| Iron and folic acid given or prescribed | 359 (79.6%) | 354 (81.9%) | 413 (90.2%) | 418 (96.1%) | 377 (86.9%) | 413 (98.8%) | 438 (89.6%) | 448 (94.9%) |
| **Completeness of all six care components**^1^ | 20 (4.4%) | 104 (24.1%) | 13 (2.8%) | 37 (8.5%) | 1 (0.2%) | 21 (5.0%) | 4 (0.8%) | 9 (1.9%) |
| **Completeness of all five care components**^2^ | 53 (11.8%) | 126 (29.2%) | 183 (39.9%) | 287 (65.7%) | 206 (47.3%) | 195 (46.7%) | 10 (2.0%) | 45 (9.4%) |

^1^ Include blood pressure measurement, blood test, urine test, ultrasound examination, iron and folic acid given or prescribed, counseling on signs of pregnancy complications

^2^ Include all the care components except for ultrasound examination.

^3^ Jodhpur: 34% urban, 66% rural; Sonipat: 31% urban, 69% rural.

**Table C. Proportion of pregnant women who received each of the six and all care components at first ANC visit in four countries by facility ownership**

| **ANC quality care components** | **Ethiopia**  **N=883** | | **Kenya**  **N=896** | | **South Africa**  **N=854** | **India**  **N=967** |
| --- | --- | --- | --- | --- | --- | --- |
|  | **Public facilities  N=635** | **Private facilities N=248** | **Public facilities  N=566** | **Private facilities N=330** | **Public facilities  N=854** | **Public facilities  N=967** |
|  | **Frequency (%)** | **Frequency (%)** | **Frequency (%)** | **Frequency(%)** | **Frequency (%)** | **Frequency(%)** |
| **Physical examination** |  |  |  |  |  |  |
| Blood pressure measurement | 416 (65.5%) | 224 (90.3%) | 556 (98.4%) | 305 (92.4%) | 852 (99.8%) | 954 (98.8%) |
| **Test and examination** |  |  |  |  |  |  |
| Blood test (blood draw or finger prick) | 596 (93.9%) | 242 (97.6%) | 553 (97.9%) | 323 (97.9%) | 853 (99.9%) | 855 (88.5%) |
| Urine test | 506 (79.7%) | 246 (99.2%) | 510 (90.3%) | 291 (88.2%) | 850 (99.5%) | 645 (66.7%) |
| Ultrasound | 141 (22.2%) | 242 (97.6%) | 53 (9.4%) | 21 (6.4%) | 65 (7.6%) | 187 (19.5%) |
| **Counseling** |  |  |  |  |  |  |
| Signs of pregnancy complications | 150 (23.6%) | 123 (50.2%) | 418 (74.1%) | 126 (38.2%) | 420 (49.4%) | 143 (14.9%) |
| **Preventions** |  |  |  |  |  |  |
| Iron and folic acid given or prescribed | 531 (83.6%) | 182 (73.4%) | 542 (95.9%) | 289 (88.1%) | 790 (92.7%) | 886 (92.2%) |
| **Completeness of all six care components**^1^ | 33 (5.2%) | 91 (36.7%) | 39 (6.9%) | 11 (3.3%) | 22 (2.6%) | 13 (1.3%) |
| **Completeness of all five care components**^2^ | 86 (13.5%) | 93 (37.5%) | 373 (65.9%) | 97 (29.4%) | 401 (47.0%) | 55 (5.7%) |

^1^ Include blood pressure measurement, blood test, urine test, ultrasound examination, iron and folic acid given or prescribed, counseling on signs of pregnancy complications

^2^ Include all the care components except for ultrasound examination.

**Table D. Results of mixed-effect logistic regressions for fetal losses and LBW newborns (ANC quality as a continuous score)**

|  | **Models for fetal losses** | | | | | | **Models for low birth weight newborns** | | | | | |
| --- | --- | --- | --- | --- | --- | --- | --- | --- | --- | --- | --- | --- |
|  | **N = 1195** | | | **N = 940**  **(among cases with reliable GA dating)** | | | **N = 3334** | | | **N = 2956**  **(among cases with actual birthweight data)** | | |
|  | Odds Ratio (OR) | 95%  Confidence Interval (CI) | *p*-value | Odds  Ratio (OR) | 95%  Confidence Interval (CI) | *p*-value | Odds Ratio (OR) | 95%  Confidence Interval (CI) | *p*-value | Odds  Ratio (OR) | 95%  Confidence Interval (CI) | *p*-value |
| **ANC quality score (%)***^1^* | 0.38 | 0.08 – 1.85 | 0.230 | 0.52 | 0.09 – 2.93 | 0.458 | 0.78 | 0.50 – 1.20 | 0.259 | 0.79 | 0.43 – 1.43 | 0.434 |
| **Socioeconomic and demographic status** |  |  |  |  |  |  |  |  |  |  |  |  |
| *Country* |  |  |  |  |  |  |  |  |  |  |  |  |
| Ethiopia | *ref* |  |  | *ref* |  |  | *ref* |  |  | *ref* |  |  |
| Kenya | 0.91 | 0.49 – 1.71 | 0.775 | 1.22 | 0.61 – 2.44 | 0.584 | 1.01 | 0.57 – 1.77 | 0.975 | 1.15 | 0.65 – 2.04 | 0.637 |
| South Africa | 0.59 | 0.18 – 1.98 | 0.395 | 0.69 | 0.19 – 2.49 | 0.569 | **1.76** | **1.25 – 2.47** | **0.001** | 1.99 | 1.53 – 2.58 | **<0.001** |
| India | 1.24 | 0.73 – 2.13 | 0.424 | **1.60** | **1.25** – **2.04** | **<0.001** | **2.00** | **1.28 – 3.10** | **0.002** | 2.35 | 1.61 – 3.42 | **<0.001** |
| *Age category. years* |  |  |  |  |  |  |  |  |  |  |  |  |
| 15 to <20 | *ref* |  |  | *ref* |  |  | *ref* |  |  | *ref* |  |  |
| ≥20 to <35 | 0.67 | 0.42 – 1.07 | 0.097 | **0.46** | **0.25 – 0.84** | **0.011** | **0.80** | **0.69 – 0.93** | **0.004** | 0.79 | 0.70 – 0.89 | **<0.001** |
| ≥35 | 1.37 | 0.39 – 4.86 | 0.623 | 0.95 | 0.35 - 2.55 | 0.919 | 1.06 | 0.70 – 1.58 | 0.792 | 1.01 | 0.72 – 1.42 | 0.968 |
| *Intended pregnancy* | 0.90 | 0.48 – 1.70 | 0.749 | 0.97 | 0.41 – 2.26 | 0.937 | 1.16 | 0.80 – 1.67 | 0.433 | 1.24 | 0.84 – 1.83 | 0.278 |
| *Education level* |  |  |  |  |  |  |  |  |  |  |  |  |
| No education or some primary | *ref* |  |  | *ref* |  |  | *ref* |  |  | *ref* |  |  |
| Complete primary | 1.16 | 0.64 – 2.09 | 0.627 | 1.59 | 0.54 – 4.69 | 0.402 | **1.40** | **1.04 – 1.88** | **0.025** | 1.26 | 0.83 – 1.93 | 0.283 |
| Complete secondary or higher | 1.06 | 0.54 – 2.08 | 0.866 | 1.49 | 0.45 – 4.93 | 0.517 | 1.02 | 0.76 – 1.36 | 0.895 | 0.94 | 0.68 – 1.29 | 0.693 |
| *Health literacy^2^* | 1.61 | 0.92 – 2.82 | 0.092 | 1.44 | 0.79 – 2.61 | 0.235 | 0.87 | 0.74 – 1.03 | 0.098 | 0.95 | 0.79 – 1.13 | 0.541 |
| *Wealth* |  |  |  |  |  |  |  |  |  |  |  |  |
| Poorest | *ref* |  |  | *ref* |  |  | *ref* |  |  | *ref* |  |  |
| Middle | 0.85 | 0.58 – 1.25 | 0.409 | 0.69 | 0.37 – 1.29 | 0.248 | 0.97 | 0.67 – 1.41 | 0.87 | 1.02 | 0.67 – 1.56 | 0.911 |
| Richest | 0.84 | 0.66 – 1.06 | 0.141 | 0.67 | 0.38 – 1.20 | 0.175 | 0.82 | 0.47 – 1.42 | 0.483 | 0.90 | 0.51 – 1.61 | 0.728 |
| **Trimester of the first ANC visit** |  |  |  |  |  |  |  |  |  |  |  |  |
| First trimester*^3^* |  |  |  |  |  |  | *ref* |  |  | *ref* |  |  |
| Second trimester*^3^* |  |  |  |  |  |  | 0.97 | 0.81 – 1.16 | 0.723 | 1.00 | 0.86 – 1.16 | 0.992 |
| Third trimester*^3^* |  |  |  |  |  |  | 0.82 | 0.42 – 1.60 | 0.557 | 0.74 | 0.34 – 1.59 | 0.433 |
| **Maternal health** |  |  |  |  |  |  |  |  |  |  |  |  |
| *Self-rated own health as very good or excellent^4^* | 1.06 | 0.59 – 1.90 | 0.849 | 1.04 | 0.49 – 2.21 | 0.909 | 0.85 | 0.67 – 1.08 | 0.194 | 0.87 | 0.63 – 1.21 | 0.412 |
| *Risk factor at baseline^5^* |  |  |  |  |  |  |  |  |  |  |  |  |
| No risk factor | ref |  |  |  |  |  | *ref* |  |  | *ref* |  |  |
| One risk factor | 1.44 | 0.90 – 2.30 | 0.131 | 1.76 | 0.91 – 3.38 | 0.091 | 1.18 | 0.96 – 1.45 | 0.124 | 1.14 | 0.84 – 1.54 | 0.409 |
| Two risk factors | 1.46 | 0.64 – 3.31 | 0.365 | 1.67 | 0.73 – 3.80 | 0.225 | 1.19 | 0.77 – 1.82 | 0.440 | 1.26 | 0.86 – 1.85 | 0.228 |
| Three or more risk factors | **2.62** | **1.01 – 6.83** | **0.049** | **2.97** | **1.05 – 8.42** | **0.040** | 0.90 | 0.39 – 2.08 | 0.803 | 1.04 | 0.44 – 2.49 | 0.924 |
| *Reported any pregnancy danger sign at baseline^6^* | **1.92** | **1.28 – 2.88** | **0.002** | **1.66** | **1.04 – 2.65** | **0.032** | 0.83 | 0.56 – 1.21 | 0.331 | 0.73 | 0.53 – 1.01 | 0.054 |

^1^ ANC quality score was measured by a continuous score ranging from 0 to 100%, reflecting the proportion of essential items out of the six received by pregnant women.

^2^ Defined as answering six health knowledge questions correctly. The six questions were adopted from the Indian Health and Human Development Survey.

^3^ The first trimester is defined as less than 13 weeks of gestation; the second trimester is defined as between 13 and less than 28 weeks of gestation; the third trimester is defined as above 28 weeks of gestation.

^4^ Self-rated own health was based on a Likert scale of five levels (excellent, very good, good, fair, and poor)

^5^ Risk factors at baseline include any chronic systemic illness(es) known before pregnancy (including diabetes, hypertension, cardiac disease, HIV, mental health disorder, schizophrenia, epilepsy, seizure, renal disorder, asthma, tuberculosis, anemia, hemoglobinopathy, chronic pelvic inflammatory disease, ovarian cyst, fibroids, uterine myoma, genital tract abnormalities, thyroid cancer, thyroid disease, peptic ulcer disease, gestational hypertension in previous pregnancy, a history of stroke), any history of obstetric complications (Cesarean section, stillbirth, preterm birth, neonatal death, and postpartum hemorrhage), and multiple pregnancy known at the first ANC visit

^6^ Pregnancy danger signs at baseline include vaginal bleeding, fever, and fainting or loss of consciousness.
