# Supplementary material for: Quality of first antenatal care visits and perinatal outcomes: Evidence from a cohort study in Ethiopia, Kenya, South Africa, and India
Source: PLOS Glob Public Health. 2026 Apr 13;6(4):e0006248. doi: 10.1371/journal.pgph.0006248 (PMC13075702; doi:10.1371/journal.pgph.0006248)
Supplement: Attachment — Submitted filename: Response letter_2025.09.30.docx [file pgph.0006248.s002.docx]

**September 30, 2025**

**RESPONSE TO REVIEWERS**

**Title:** Quality of antenatal care and perinatal outcomes: evidence from a cohort study in Ethiopia, Kenya, South Africa, and India

Dear Editor-in-Chief,

We are pleased to submit a revised version of our manuscript titled "Quality of antenatal care and perinatal outcomes: evidence from a cohort study in Ethiopia, Kenya, South Africa, and India" to **PLOS Global Public Health**. **This paper was reviewed by three reviewers invited by PLOS Medicine. We have addressed these reviewer comments and are now transferring this paper to PLOS Global Public Health.**

We would like to thank the reviewers for their careful critique of our manuscript, which has helped improve the study. We provided point-by-point responses to the reviewers' comments below, and corresponding changes have been made with track changes in the attached manuscript.

We hope that the revised article will be of interest to your journal.

Sincerely,

Catherine Arsenault, PhD, MSc, on behalf of all co-authors

Assistant Professor | Global Health and Health Systems
Milken Institute School of Public Health
The George Washington University
**Tel:** 202-994-1011
[catherine.arsenault@gwu.edu](mailto:email@gwu.edu)

**Responses to the reviewers' comments**

| **Reviewer 1 comment** | **Author response** |
| --- | --- |
| 1. Page 10 I appreciate we could look at the three cited references to obtain more details but it would be helpful to have more details about how the women were selected into the cohort. Is this a complete enumeration of pregnant women in the study site? Elsewhere there is a hint that there was more than one health care facility in each of the eight sites. Is that true? | We appreciate your comment. There were between 21 to 29 facilities in each country that joined this study. We added sentences about the number and type of facilities in line 148 to 159.  Our protocol also specified that a number of 500 pregnant women per site were aimed to enroll. We also added relevant details in line 159 to 160.  Line 148 to 159  The study was conducted at two sites (one urban, one rural) in each country. Between 21 and 29 facilities were selected for inclusion in each country. The health facilities selected included 1) both public and private primary and secondary facilities in Ethiopia and Kenya, 2) only public primary and secondary facilities in India, and 3) only public primary facilities in South Africa. Pregnant women aged 15 and above (or 18 and above in India), at any gestational age, who presented to these facilities to have their first ANC visit, planning to give birth in the study site and consenting to participate were enrolled in this study.  Line 159 to 160  The MNH eCohort study aimed to enroll 500 pregnant women per site in each county, with a minimum of 50 women per facility strata. |
| 2. Page 11 Do we have any information, either from this study or elsewhere, as to the reliability of the mother's assessment of their newborn's weight? | Thank you. Our survey was designed to ask all women with live babies the following two questions: 1) their baby's or babies' weight at birth, and 2) whether the baby was very large, larger than average, average, smaller than average, or very small when the baby was born. We used actual newborn birth weight to determine low birthweight; if the birthweight data were unavailable, we then used mother-reported size. The approach of mother self-reported baby birth size was adapted by DHS surveys, while studies showed varied levels of accuracy. (1, 2, 3)  1 newborn? Assessing the determinants of a mother's perception of a baby's size at birth. J Biosoc Sci. 2011 Sep;43(5):555-73.. Channon AA. Can mothers judge the size of their  2. Nigatu D et al. Predictive accuracy of perceived baby birth size for birth weight: a cross-sectional study from the 2016 Ethiopian Demographic and Health Survey. BMJ Open. 2019 Dec 11;9(12):e031986  3. Acharya P et al.Evidence from Nepal Demographic and Health Survey. PLoS One. 2023 Jan 24;18(1):e0280788  In our discussion session, we also acknowledged the limitation that "the LBW analysis was limited by the mother's self-reported baby birth size, with only 60% of babies in Ethiopia having actual birth weight data, compared to 90% in the other three countries." in line 414 to 416. |
| 3. Page 11 and 12 The authors have reduced the six variables of interest into two alternative binary variables. I found that rather surprising. Why not treat each separately? Or put all five or six into one overall model to see the associations individually? | Thanks for this question. The five-six items were transformed into binary indicator to reflect the health system's ability to provide women with a full package of essential services at the 1^st^ ANC visit. Previous analyses have used binary indicators to measure ANC quality ([Arsenault et al. The Lancet Global Health, 2018](https://www.thelancet.com/journals/langlo/article/PIIS2214-109X(18)30389-9/fulltext)). In other words, getting only one or a few of these essential care items does not represent high quality care. In addition, the effect on perinatal outcomes likely comes from comprehensive patient assessments, adequate management of comorbities and danger signs and continuous follow up throughout pregnancy. These dimensions of quality are likely linked to receiving the full package of essential care during ANC1 not only specific items separately.  Nonetheless, to respond to the reviewer's comment, we added a third sensitivity analysis where ANC quality is measured by a continuous score ranging from 0 to 100% and reflecting the proportion of items out of the 6 recommended received by the woman. Please refer to the relevant changes in line 253 to 255 in the method section, and line 327 to 329 in the result section and supplemental Table 4.  We found that a one percentage point increase in ANC1 content was associated with 22% and 62% lower odds of LBW and fetal losses, respectively. However these estimates were not statistically significant.  Line 253 to 255  Third, we run models where ANC quality was measured by a continuous score ranging from 0 to 100%, reflecting the proportion of essential items out of the six received by pregnant women.  Line 327 to 329  Regressions using ANC quality as a continuous score did not show significant associations with continuous scores and both outcomes of interest (Supplemental Table 4). |
| 4. Page 13 Does the word included imply that there were in fact more than the four enumerated here? The fifth footnote to Table 1 suggests there were more. In that case I would list them all here in the methods. I am not a great fan of collapsing categorical variables but the frequencies in Table 1 suggests that it was a sensible decision here. | Thank you for this comment. In line 219 to 222, we described the risk factors assessed, which included 1) presence of any chronic illness(es) diagnosed before the pregnancy, 2) a history of obstetric complications (including Cesarean sections, preterm birth, stillbirth, neonatal death, or postpartum hemorrhage), 3) HIV, and 4) multiple pregnancy detected at the first ANC visit. Similarly, the fifth footnote in Table 1 also describes the same list of risk factors, with all the illnesses included in any chronic illness listed. |
| 5. Page 13 With only two sites per country fitting a random intercept seems quite ambitious. Why not fit a fixed effect for rural/urban? | We used two levels of random intercepts (site and facility) to account for the clustering at both levels and to improve variance estimation. The site-level random intercept includes eight distinct sites (two sites per country), not rural/urban classification. The context of rural or urban regions across four countries could vary a lot; therefore, using rural/urban classification might not reflect the variations across eight sites. |
| 6. Page 13 I do not use Stata so I only have a vague idea what a post–estimation command is. I assume these are what R calls methods. A more software–agnostic term would be preferable. | Thank you. We have made relevant edits. Please see line 231 to 233.  Line 231 to 233  We used predictions from the fitted models to calculate marginal probabilities and obtained risk ratios for the association between good quality ANC and each primary outcome. |
| 7. Page 15 The range quoted here is presumably not the range of the mothers' ages. Perhaps clarify what is meant? It might be simpler just to quote the extremes. | Thank you, and we agreed that the original wording is not clear. We have made relevant edits in line 271 to 272.  Line 271 to 272  The mean age of participants was 25.9 years old (mean age 24.5 in India to 26.9 in Kenya). |
| 8. Page 15 Here we discover for the first time (as far as I can see) that multiple births were included. How was that taken into account in the models? Were both children included in the low birth weight analysis? Is that valid for multiple births? I doubt whether there is a right answer here but we do need to know what the authors chose to do. | Thank you. We explored options around this issue when developing our analysis plan. Some published studies did include both babies in twin pregnancies or all babies from multiple pregnancies. This is what we meant by "at the neonatal level." We have added sentences to clarify this in line 234 to 235.  Line 234 to 235  Regressions were performed at the fetal and neonatal level, with all eligible fetuses/babies from multiple pregnancies included separately. |
| 9. Page 17 We need the effect sizes for the ones which did not reach the arbitrary level of statistical significance. | For brevity, effect sizes of other covariates are reported in Table 4 (page 34) |
| 10. Table 2 The fact that some of these are nearly always present suggests that differences in quality may be driven by only some of the components. Has that been considered? This is another reason to consider models taking the components separately. | Thank you for your important comment on this. Our hypothesis is that comprehensive ANC quality meeting basic minimum standards will improve perinatal outcomes, not specific items in particular. The reason for splitting out ultrasounds in a sub-analysis is that they are not always consistently recommended and available in rural settings. All other items should be performed even in the lowest resourced settings.  To further explore how exposure specification may influence the findings, we have added an additional sensitivity analysis where we modelled ANC quality as a continuous score for the proportion of 6 items completed. Please see reviewer comment #3 and supplemental table 4. |
| 11. Supplementary Table 1 It might be helpful to flag which is the rural site and which the urban. Having grown up in what in my country counts as a rural site I assume the ones on the left of each pair are rural since  we always get poorer services. | Thank you. We made relevant edits to the Supplemental Table 2. |
| 12. Immortal time bias. I agree with the sentiments expressed on page 12 about this but they do not seem to me to apply to the analysis of low birth weight. To be recorded as having a birth weight I assume that the child has to arrive at term or fairly close to it. In that case the quality of care data could take account of what happened at all visits not just the first. This might help to answer some of the questions raised on page 18 about the role of ultrasound. If policy leads to it only being considered later in pregnancy then its relative absence at baseline is understandable and not perhaps an indicator of poor care. | Thank you for this comment. However, the definition for LBW is a weight of less than 2500g. Although some of these babies are born at term, many LBW babies will be born premature (at 24 to 37 weeks' gestation) and therefore, many women giving birth to these LBW babies have a much shorter pregnancy with fewer opportunities to get additional care. Including care recorded in follow-up surveys in our quality measure could bias the findings since some women experiencing the outcome did not have follow up surveys. This is especially true considering that the majority of women initiated ANC and were enrolled in the study in the second trimester (between 13 and 28 weeks of gestation). |
| 13. Interactions with country. Given the fairly substantial differences between the countries revealed in the tables it would be possible to see whether the components of care had different effects in the four countries. | Thank you for this important comment. We did discuss this internally; however, given the low frequency of our outcomes in each country, we were concerned about losing power in models with rare outcomes. |
| 14. Variable selection. The different list of variables for the two models in Table 4 and Supplement Table 3 suggests variable selection has taken place. This should have been mentioned in the methods. Selecting a subset of variables in a way driven by the data leads to a model which is unlikely to replicate (Babyak, 2004). Screening variables to enter the model has been criticised too (Sun et al., 1996) I suggest all the models should use the full set of covariates.  I would also question the positioning of the low birth weight model in the supplement. This was one of the two chosen outcomes. Just because it did not seem to be related to quality of care is no reason to shunt it off into the supplement. It is also rather downplayed in the abstract. | We really appreciate this comment. For your information, the only difference in covariates between the fetal loss and LBW models is the adjustment for trimester at ANC initiation. We did not adjust for trimester at ANC initiation in the fetal loss models because they were restricted to women who initiated in their first trimester. All other covariates are the same, and this is mentioned in the statistical analysis section in the methods (Please see Table 4)  Meanwhile, we agree with your second comment and have moved the LBW model results to the new Table 4 (containing results from fetal loss models and LBW models) in the main paper. Please see the relevant changes in Table 4 (page 34). |
| **Reviewer 2 comments** | **Author response** |
| 1. Introduction. The introduction effectively emphasizes the importance of quality antenatal care. Including references to relevant studies from specific geographies would strengthen the context. Additionally, some ideas, such as those on lines 113 and 121, appear repetitive and could be streamlined for clarity. | Thank you. We have made relevant edits to make it more concise. Please refer to line 115 to 124. |
| 2. The study design is commendable for its complexity and thorough follow-up from early pregnancy to three months postpartum. However, additional details on the study methods would enhance clarity and reproducibility. Specifically, please clarify the total number of health facilities involved, and site inclusion criteria and level of the facilities.  Reading the referenced study protocol, it mentioned 2 sites were selected per country, one urban and one rural but not the number of health facilities in total. I read that higher level facilities were excluded as first booking visits are only accepted at lower level health facilities. | Thank you. We have made relevant edits to the methods section. Please refer to line 148 to 159.  Line 149 to 159  The study was conducted at two sites (one urban, one rural) in each country. Between 21 and 29 facilities were selected for inclusion in each country. The health facilities selected included 1) both public and private primary and secondary facilities in Ethiopia and Kenya, 2) only public primary and secondary facilities in India, and 3) only public primary facilities in South Africa. Pregnant women aged 15 and above (or 18 and above in India), at any gestational age, who presented to these facilities to have their first ANC visit, planning to give birth in the study site and consenting to participate were enrolled in this study. |
| 3. Kindly consider adding some additional contextual description of the study area, the population, and facility births in that geography. | Thank you. As the current manuscript has exceeded 4100 words, for brevity and readability, we added details on the facilities that were included and provided references (our study protocol and a study we published before) for readers to refer to. |
| 4. The sample size calculation and handling of missing data, loss to follow-up, and exclusions (e.g., uncertain gestational age) require more detailed explanation | Thank you. Similarly, the sample size calculation was provided in our protocol. We have also added about how missingness was handled in line 256 to 257.  Line 256 to 257  Analyses were conducted using complete data, excluding cases with missing values. |
| 5. Line 225-226: Regressions were performed at the fetal and neonatal level. The first model assessed associations between good quality ANC and fetal losses (late miscarriages or stillbirths). Regarding analyses, please clarify the term "neonatal level" in the regression models and confirm whether neonatal deaths were assessed. | Thank you. We explored options around this issue when developing our analysis plan. Some published studies did include both babies in twin pregnancies or all babies from multiple pregnancies. This is what we meant by "at the neonatal level." We have added sentences to clarify this in line 234 to 235.  Line 234 to 235  Regressions were performed at the fetal and neonatal level, with all eligible fetuses/babies from multiple pregnancies included separately. |
| 6. Line 227- The fetal loss regression was restricted to women who were enrolled in their first trimester of pregnancy to avoid selection bias. Specify how many women were excluded from the fetal loss regression due to enrolment timing in the first trimester (line 227) | Thank you. In our table column 2, we showed that only 33.3% of participants were enrolled in their first trimester. |
| 7. Would the authors consider adding some explanation regarding the inclusion criteria (during analysis). It appears that the study included only the first antenatal visit and then for some outcomes, only women who booked in the first trimester were included and again later the manuscript mentions that only those with evidence of reliable gestation age were included. Given the complex inclusion criteria around gestational age and first ANC visit, a flow chart could greatly aid reader's understanding. | Thank you. The study enrolled women during their first ANC visit regardless of gestational age. Women were followed until they gave birth. However, due to the nature of our outcomes, our regression models used subsets of respondents. We ran models for two primary outcomes: one for fetal losses (any fetal loss from 13 weeks of gestation and until delivery) and one for LBW newborns.  1) For models of fetal losses, we only included pregnant women who were enrolled in the first trimester (before 13 weeks) in order to avoid immortal time bias, which was explained in lines 236 to 241. The sensitivity analysis for this model was to restricct the sample to women with more reliable GA assessment at enrollment.  2) For the LBW regression, we included all women regardless of GA at enrollment. The sensitity analysis for this model was to restrict the sample to those that had more reliable birth weights.  For clarity, we have made edits in the methods and added a Supplemental Table 1 to describe the models. |
| 8. A minor point: gestational age estimation is typically derived from the LMP, which is used to estimate the EDD. At best, EDD from LMP can be less reliable than early ultrasound-based dating and should be noted. Additionally, for the LBW sensitivity analysis, consider that birthweight interpretation should mention the limitation of poor gestational age dating that limits distinguishing small-or-large-for-gestational-age from appropriate-for-gestational-age infants. | Thank you for this comment. We have mentioned limitations around our birthweight data in line 415 and 417. |
| 9. Line 297 describes 'Risk ratios (RR) for the association between good quality ANC and perinatal outcomes from the eight regression models are summarized in Figure 1. The methods section would benefit from more detailed explanations of the regression models and their outputs to enhance clarity for both researchers and practitioners. | Thank you. We have made edits in line 309 to 316 to make it clearer. |
| 10. Results. Would the authors please clarify whether Table 3 includes neonatal deaths, and explain the discrepancy between the number of live births and those included in the neonatal outcomes row?  Additionally, the meaning of footnote 7 is unclear and would benefit from further explanation. | Thank you. In the method section, we mentioned in line 182 to 184 that data on birth body weight or mother-reported birth body size were only collected for newborns who were still alive at the time of the final follow-up phone survey. We also included this point as one of our limitations in line 413 to 417. In other words, neonatal deaths, born alive but not alive at post-delivery surveys, were not included in the sample for LBW regressions.  For the comment on footnote 7, we have edits to make it clearer (current footnote 8 in table 3). |
| 11. In Figure 1, please specify the origin of the n=1194 participants included in the primary analysis. | Thank you. We had footnotes explaining the models and samples, but agreed with you that we should specify the sample. Please see the edited footnote.  The samples for fetal loss regressions came from 1) the fetuses with an outcome of late miscarriage, stillbirth, or livebirth, resticted to those whose mothers enrolled in the first trimester in primary analysis, and 2) the fetuses with an outcome of either late miscarriage, stillbirth, or livebirth, resticted to those whose mothers enrolled in the first trimester and had more reliable gestational age dating in secondary analysis. The samples for LBW regressions came from 1) the neonates who were alive at post-delivery surveys in primary analysis, and 2) the neonates who were alive at post-delivery surveys and had actual birthweight data in secondary analysis. |
| 12. The discussion emphasizes the importance of quality antenatal care, early ultrasound, and multiple pregnancy. Figure 1 suggests that including ultrasound in the quality package reduced miscarriages and stillbirths by 58% (compared to 32% without ultrasound). The Uss effect cannot be overlooked even though the small sample size and potential confounders warrant cautious interpretation. A deeper analysis of the sociodemographic factors influencing early ultrasound access could be valuable, along with a clearer consideration of how an early ultrasound might directly impact clinical outcomes in the third trimester. | Thank you. We do agree that this is important; however, the main focus of this study is to assess the relationship between receiving essential care components and fetal/neonatal outcomes, rather than how sociodemographic factors affect access to care. We would continue exploring the area you suggested in our future research. |
| 13. Some sections could benefit from reducing repetition and refining terminology—for example, using "babies in breech position" rather than "breeched babies," and clarifying that obstructed labor is a clinical, not ultrasound, diagnosis. Highlighting the main findings, in relation to recent LMIC literature, including studies from Ethiopia and Tanzania etc., on danger signs in late pregnancy and perinatal losses, would enhance the discussion. | Thank you for this comment. We have made relevant edits in line 345 to 346. |
| 14. Please also review the formatting of in-text references and punctuation placement. | Thank you. We have made relevant changes to comply with the journal's requirements. |
| **Reviewer 3 comments** | **Author response** |
| It is somewhat surprising that for India no private facilities are included, given that more than half of facilities are not public. Why is this the case and what does it mean for generalizability? And how does this link to the notion that "When excluding private facilities, ANC quality ranged from 1.4% in India ...", where there any private facilities in India to exclude? | Thank you. The initial design regarding facility selection was determined by multiple factors, including available resources, willingness and readiness to participate, etc. We agree with you on the issue of the limited generalizability of our findings, and clarified these limitations in line 425 to 427.  Line 422 to 424  Lastly, we acknowledge the limited generalizability of our findings, given that the study was conducted in selected facilities within two particular sites in each country. |
| **Reviewer 4 comments** | **Author response** |
| 1. The authors mention that there are limited studies on this topic. However, there are several studies from Africa and Asian countries that examine the relationship between quality ANC and perinatal outcomes. How does your study differ from these existing studies? It would be helpful to clarify this point. | Thank you. In the introduction and discussion sections, we described the differences and similarities between previous studies and our findings in lines 115 to 117 and line 375 to 377. |
| 2. The statement "Our study found that ANC quality was overall poor" requires clarification. How was the concept of "poor" ANC defined and measured? Please specify the criteria to make this determination. | Thank you, and we agreed that this is crucial. In line 192 to 208 of the method section, we clearly defined good quality ANC, which is a binary indicator capturing the essential components that pregnant women should receive. |
| 3. The terms "neonatal outcomes" and "fetal loss" appeared to be used interchangeably. I recommend choosing one term or clearly distinguishing between the two for consistency and accuracy. | Thank you. We have cross-checked the manuscript to ensure that the term "fetal loss" was used to refer to late miscarriages and stillbirth, and the term "neonatal outcome" was used to mean outcomes among neonates generally. |
| 4. The classification of the five and six care component needs further explanation. What criteria or rationale did the authors use to define these categories? Needs references. | Thank you. We provided our rationale for selecting these six components based on the WHO recommendations and previous studies on ANC quality in line 197 to 198. We also cited supporting references. |
| 5. The term "good clinical practice" may not be appropriate here, as many aspects of ANC have not been addressed in the study. It would be clearer to specify whether the quality issues are related to provider practice, system-level factors or resource constraints. | Thank you. We made relevant edits in line 66 and line 93. |
| 6. Line 122 requires appropriate references. | We checked our submitted manuscript, and the relevant references were cited in line 122. Please see line 121 to 124 in the original submitted version.  Line 121 to 124  Hence, high quality ANC is crucial for addressing these risk factors. Few studies have assessed associations between ANC quality and stillbirths. One study from Ghana found an association between good quality ANC and a reduced risk of stillbirth (8); another study in Nairobi, Kenya, also demonstrated the protective effects of good quality ANC against stillbirth.(7)  Ref 7. Gwako GN et al. Association between utilization and quality of antenatal care with stillbirths in four tertiary hospitals in a low-income urban setting. Acta obstetricia et gynecologica Scandinavica. 2021;100(4):676-83. Epub 2020/07/11.  Ref 8. Afulani PA. Determinants of stillbirths in Ghana: does quality of antenatal care matter? BMC pregnancy and childbirth. 2016;16(1):132. Epub 2016/06/04. |
| 7. The statement Delivering high-quality ANC remains a challenge in LMIC settings, with the highest burdens of maternal and newborn morbidity and mortality" should be supported by a reference. Also, considering that the study did not examine the health system or other contextual barriers affecting ANC utilisation. | Thanks for the comment. We have added relevant citations to support this statement. Please refer to line 429. |
