# Supplementary material for: Quality of first antenatal care visits and perinatal outcomes: Evidence from a cohort study in Ethiopia, Kenya, South Africa, and India
Source: PLOS Glob Public Health. 2026 Apr 13;6(4):e0006248. doi: 10.1371/journal.pgph.0006248 (PMC13075702; doi:10.1371/journal.pgph.0006248)
Supplement: Attachment — Submitted filename: Response ro reviewer_2026.03.06.docx [file pgph.0006248.s003.docx]

**March 6, 2026**

**RESPONSE TO REVIEWERS**

**Title:** Quality of first antenatal care visits and perinatal outcomes: evidence from a cohort study in Ethiopia, Kenya, South Africa, and India

Dear Editors,

We are pleased to submit a revised version of our manuscript titled "Quality of antenatal care and perinatal outcomes: evidence from a cohort study in Ethiopia, Kenya, South Africa, and India" to **PLOS Global Public Health**. We have provided point-by-point responses to the reviewers' comments below, and corresponding changes have been made in track changes.

We would like to thank the reviewers for their careful critique of our manuscript, which has helped improve the study. We hope that the revised article will be of considerable interest to the broad scientific, clinical, and policy experts in the field of improving global newborn health.

Sincerely,

Catherine Arsenault, PhD, MSc, on behalf of all co-authors

Assistant Professor | Global Health and Health Systems
Milken Institute School of Public Health
The George Washington University
**Tel:** 202-994-1011
[catherine.arsenault@gwu.edu](mailto:email@gwu.edu)

**Responses to the editor's and reviewers' comments**

| **Editor comment (Journal requirement)** | **Author response** |
| --- | --- |
| 1. Please provide a detailed online Financial Disclosure statement. This is published with the article. It must therefore be completed in full sentences and contain the exact wording you wish to be published.  a) State the initials, alongside each funding source, of each author to receive each grant. For example: "This work was supported by the National Institutes of Health (####### to AM; ###### to CJ) and the National Science Foundation (###### to AM)."  b) State what role the funders took in the study. If the funders had no role in your study, please state: "The funders had no role in study design, data collection and analysis, decision to publish, or preparation of the manuscript." | Thank you. We have added the statement to line 433.  *Line 433*  Funding for the eCohort study was obtained from the Bill and Melinda Gates Foundation (grant number INV-005254 to MEK) and the Swiss Federal Department of Foreign Affairs (grant number 81067262 to MEK). The funders had no role in study design, data collection and analysis, decision to publish, or preparation of the manuscript. |
| 2. Please ensure that the funders and grant numbers match between the Financial Disclosure field and the Funding Information tab in your submission form. Note that the funders must be provided in the same order in both places as well. | Thank you. See above. |
| 3. Please update your online Competing Interests statement. If you have no competing interests to declare, please state: "The authors have declared that no competing interests exist." | Thank you. |
| 4. In the online submission form, you indicated that your data will be submitted to a repository upon acceptance. We strongly recommend all authors deposit their data before acceptance, as the process can be lengthy and hold up publication timelines. Please note that, though access restrictions are acceptable now, your entire minimal dataset will need to be made freely accessible if your manuscript is accepted for publication. This policy applies to all data except where public deposition would breach compliance with the protocol approved by your research ethics board. If you are unable to adhere to our open data policy, please kindly revise your statement to explain your reasoning and we will seek the editor's input on an exemption. | The analytic data is available at: <https://dataverse.harvard.edu/dataset.xhtml?persistentId=doi:10.7910/DVN/Q0YKOT> |
| 5. Please provide separate main figure files in .tif or .eps format only and ensure that all files are under our size limit of 10MB. For more information about how to convert your figure files please see our guidelines:  <https://journals.plos.org/globalpublichealth/s/figures> | Thank you. We have submitted a separate figure. |
| 6. If the reviewer comments include a recommendation to cite specific previously published works, please review and evaluate these publications to determine whether they are relevant and should be cited. There is no requirement to cite these works unless the editor has indicated otherwise. | Thank you for letting us know. |
| 7. Please review your reference list to ensure that it is complete and correct. If you have cited papers that have been retracted, please include the rationale for doing so in the manuscript text, or remove these references and replace them with relevant current references. Any changes to the reference list should be mentioned in the rebuttal letter that accompanies your revised manuscript. If you need to cite a retracted article, indicate the article's retracted status in the References list and also include a citation and full reference for the retraction notice. | Thank you. We have checked the formatting of references. |
| 8. As you will see from the reviewers written comments, some suggestions are substantive and others are stylistic. As you are preparing your revision, please pay attention to the substantive comments (for example, reviewer #2's comments about making clear that quality is based on the first antenatal care visit) but feel free to use your discretion as you review the more stylistic edits requested. | Thank you. We have prepared our revised manuscript accordingly. |
| **Reviewer 1 comments** | **Author response** |
| 1. We appreciate the team and collaborative effort for the study. The guideline of the journal should be used well. E.g. Methods and findings in the abstract sub section are not in line with the guideline of the journal. The format is incorrect. | Thank you. We have changed the format to ensure it meets the journal requirements. We also revised the abstract to be within 300 words. |
| 2. Avoid general words E.g. In line 37-38 In some settings., Again line 39 resource limited countries | Thank you. We have made changes to line 37. The sentence in line 39 was removed due to the word count limit.  *Line 37*  Although ANC coverage has improved globally, ANC quality remains suboptimal in low- and middle-income countries. |
| 3. Grammar issues E.g. Line 39 can be rewritten as This study had assessed | Thank you. As some parts in the first paragraph were removed, we currently keep that relevant sentence as it is now. |
| 4. Inconsistency E.g. perinatal outcomes Versus fetal loss and LBW | Thank you. We have made changes to explain that perinatal outcomes included both fetal loss and LBW in lines 44-45.  *Lines 44-45*  Perinatal outcomes of interest included fetal losses (≥13 weeks of gestation) and LBW newborns. |
| 5. Some lack full stop and contain incomplete sentence | Thank you. We have reviewed the manuscript and ensured that all sentences are complete. |
| 6. *Abstract* The gap is weak. Maintain consistency. The result should report confidence interval after the percentage What about Quality ANC and fetal loss? [Is there association] The conclusion is not in line with the objective of the study E.g. Quality gaps Versus quality ANC. | *RE evidence gaps*  Because of the word count limit, we did not elaborate on the evidence gap in the abstract; we discussed it further in lines 112-125 in the introduction section.  *RE reporting confidence interval*  As for the confidence intervals in the abstract, we did include CIs in lines 59-60.  *RE associations between quality ANC and fetal loss*  In lines 58-61, we stated that "Good quality ANC was associated with a 32% to 59% lower risk of fetal loss (primary analysis: RR 0.41, 95% CI 0.10 – 0.72; sensitivity analysis: RR 0.68, 95% CI 0.44 – 0.92). No significant associations were observed between good quality ANC and LBW."  *RE quality gaps*  We referred to "important gaps in ANC quality" as deficiencies in ANC quality. |
| 7. *Introduction* The section fails to contain what it should scientifically contain e.g. What are efforts made globally, regionally and nationally in the respective countries to escalate quality ANC and reduce adverse perinatal outcomes? What is the importance of stating still birth instead of fetal loss? | Thank you for your comments.  *RE efforts made globally and in the respective countries*  As the focus of this paper is to examine how ANC is linked to perinatal outcomes, we did not mention prior efforts to improve ANC quality.  *RE stillbirth instead of fetal loss*  We defined fetal loss as late miscarriages and stillbirths (lines 165-169). Therefore, we mentioned prior studies focusing on the associations between ANC and stillbirths. |
| 8*.Methods* The number of sites per country should be stated. The detail of the study participants was missed E.g. Age.  When do you say quality ANC? Is that based on the number of visits or have you used quality frameworks to assess it? If so, what type of quality framework and why? Is that ANC utilization study? Or?  If Ultrasound was used why you take the rough estimate of pregnancy by the pregnant women herself?  The primary outcome lacks reference? E.g. Low birth weight. In addition, you failed also to define the fetal loss and cite appropriate reference.  Line 191-192 We also created a good quality ANC binary variable based on the receipt of five care components (excluding ultrasound scan). Is not in line with the abstract. This is also not in line with the idea in line 248.  How do you manage data from the public health facilities and mixed data from the public and the private health facilities [Comparability and how you treated it?]  Have you used the current recommendation of WHO ANC guideline or Country guideline? Meaning what is the source of the quality ANC at six visits? What are primary independent variables? Have you done model fitness? | *RE sites and participants*  In lines 144-151, we described study sites and facilities, and the participant inclusion criteria, including age.  *RE definition of ANC quality*  In lines 185-189, we defined good quality ANC in the first visit as receiving six essential care components. As we mentioned in the introduction, prior research focused on how the number of visits is associated to adverse outcomes. We believe that the care content received during the visit is what really matters.  *RE ultrasound*  For women who received at ultrasound, the gestational age (GA) was based on the GA indicated in the maternal health card. We clarified this in the method on lines 331-335.  *331-335*  *The GA was estimated from the maternal health card where possible, using the recorded gestational age, date of last menstrual period (LMP), or estimated due date from the first ANC visit. When these were unavailable, LMP as reported by the woman was used, or, as a last resort, her self-reported weeks of pregnancy at baseline.*  *RE definition of primary outcomes*  The definition of fetal loss was provided in lines 165-169 with a reference from the WHO. The definition for LBW was provided in lines 173 and 175. We also added one reference here based on the reviewer’s comment. (Please see line 175 [32])  *RE good quality ANC definition*  The outcome variable of receiving five care components was developed for sensitivity analysis as the South African guidelines did not recommend ultrasound to all pregnant women at the time when the study was conducted.  *RE facilities*  We reported the differences in facilities enrolled explicitly in the method section. In the supplemental file, we also reported the proportion of participants receiving good quality ANC, stratified by facility type. (Please see Supplemental Table 3)  *RE WHO and country guidelines*  Thank you for the comment, we have reviewed the WHO recommendations and country guidelines, and a relevant study was previously published ([Arsenault C, et al. Antenatal care quality and detection of risk among pregnant women: An observational study in Ethiopia, India, Kenya, and South Africa. PLoS Med. 2024 Aug 27;21(8):e1004446.](https://journals.plos.org/plosmedicine/article?id=10.1371/journal.pmed.1004446)) In addition to the WHO guidelines, we also cited other references to identify these six essential components in lines 189-190. |
| *9. Results* Present systematically Sub group analysis E.g. Age Maintain coherence Use illustrative E.g For the perinatal outcome Tables are incomplete E.g Sample size. | Thank you so much for the comment. We presented our results in four sections: characteristics of study participants, ANC quality at the first visit, perinatal outcomes, and regression results, hoping that this structure can make it easy for the audience to understand.  *RE subgroup analysis by age*  As most of our participants were aged 20 to 35, we did not stratify the results by age, as the sample sizes for certain age groups might be small. |
| *10. Discussion and Other consequent sections* The discussion is weak and needs enrichment Use your own main findings Try to write strong theoretical and practical implication with appropriate explanations, justifications and reference. It lacks recommendations. | Thank you. We have revised the discussion section including the lines below and expanded the discussion on the reasons for a lack of association with LBW.  *Lines 420-430*  While access to and utilization of antenatal care have improved globally, delivering high-quality ANC remains challenging in LMICs, where the burden of maternal and newborn morbidity and mortality is highest. Future research should continue to assess ANC quality, particularly longitudinal quality over the course of pregnancy, with a focus on the appropriateness of risk stratification, disease management, and referrals. In addition, it should examine how longitudinal ANC quality affects adverse perinatal outcomes. Meanwhile, national policies should foster environments that support good clinical practices and the delivery of essential care components that define high-quality ANC. |
| **Reviewer 2 comments** | **Author response** |
| 1. The Title, Abstract its Conclusion, as well as the Discussion Section must reflect the specific exposure which is the “Quality of the First Antenatal Care Visit.” Thus, the word “First” or “Initial” should be used to specify the antenatal quality this research assessed. | Thank you for this important comment. We have made relevant changes in the manuscript, including title, abstract, and throughout the main manuscript. |
| 2. The Discussion must contain a prominent subsection explicitly detailing the implications of using a first-visit-only quality measure. The authors should state that their study design was not able to test the effect of longitudinal, ongoing care quality, which may be more relevant for outcomes like LBW. | We appreciate this comment and have added one sentence to clearly explain this in lines 404-406.  *Lines 404-406*  Thus, the current study design could not assess the associations between longitudinal ANC quality and outcomes that might occur before the full assessment of exposure. |
| 3. It can be recommended that future research should investigate how to maintain high-quality care across all ANC contacts, as this longitudinal quality may be critical for preventing outcomes like low birth weight, which this study was not designed to assess. | Thank you. We have added relevant discussions in lines 421-427.  *Lines 421-427*  While access to and utilization of antenatal care have improved globally, delivering high-quality ANC remains challenging in LMICs, where the burden of maternal and newborn morbidity and mortality is highest. Future research should continue to assess ANC quality, particularly longitudinal quality over the course of pregnancy, with a focus on the appropriateness of risk stratification, disease management, and referrals. In addition, it should examine how longitudinal ANC quality affects adverse perinatal outcomes. |
| 4. Reframe the LBW finding: We found no association between the completeness of the first ANC visit and low birth weight. This will not render subsequent antenatal care at other time points irrelevant | Thank you for this suggestion. We have made relevant changes throughout the paper.  *Lines 567-578*  *There are a few potential explanations. First, the care components we identified were proxies of good quality ANC, drawn from the first ANC visit only and may not capture all essential elements that affect LBW, including adherence to IFA supplementation for at least 90 days. Second, MMS is not yet routinely provided in the study countries, though it is likely to be scaled up in the coming years. Similarly, BEP supplementation is not universally available. Programs such as Ethiopia's targeted supplementary feeding program deliver BEP to undernourished pregnant women (30%–50% of pregnant women are undernourished in Ethiopia), but implementation has faced considerable challenges and only certain districts are targeted. Taken together, these gaps suggest that higher-quality ANC—including better integration of nutritional interventions—has the potential to further reduce LBW* |
