# Supplementary material for: Quality of first antenatal care visits and perinatal outcomes: Evidence from a cohort study in Ethiopia, Kenya, South Africa, and India
Source: PLOS Glob Public Health. 2026 Apr 13;6(4):e0006248. doi: 10.1371/journal.pgph.0006248 (PMC13075702; doi:10.1371/journal.pgph.0006248)
Supplement: Attachment — Submitted filename: Response ro reviewer_2026.03.17.docx [file pgph.0006248.s004.docx]

**March 17, 2026**

**RESPONSE TO REVIEWERS**

**Title:** Quality of first antenatal care visits and perinatal outcomes: evidence from a cohort study in Ethiopia, Kenya, South Africa, and India

Dear Editors,

We are pleased to submit a revised version of our manuscript titled “**Quality of first antenatal care visits and perinatal outcomes: evidence from a cohort study in Ethiopia, Kenya, South Africa, and India**” to **PLOS Global Public Health**.

We have provided point-by-point responses to the editor’s comments below, and corresponding changes have been made in track changes.

Sincerely,

Catherine Arsenault, PhD, MSc, on behalf of all co-authors

Assistant Professor | Global Health and Health Systems
Milken Institute School of Public Health
The George Washington University
**Tel:** 202-994-1011
[catherine.arsenault@gwu.edu](mailto:email@gwu.edu)

**Responses to the editor's and reviewers' comments**

| **Editor comment** | **Author response** |
| --- | --- |
| Please could you also add some text to the background section that explicitly highlights the importance of the first ANC visit? I think that is important now that the paper makes clear that the data collected speaks to that visit specifically. | Thank you. We expanded the background section in lines 291 to 305:  *The first ANC visit is particularly critical, as it establishes the foundation for pregnancy care by enabling early risk identification, baseline health assessments, and timely initiation of preventive interventions. [1] Global and national ANC guidelines recommend a core set of services during the first ANC content: blood pressure measurement, blood and urine testing, screening for infections such as HIV and syphilis, iron and folic acid supplementation, counseling on pregnancy danger signs and birth preparedness, and risk stratification to determine whether the woman requires routine or specialized care. An early obstetric ultrasound before 24 weeks of gestation is also recommended by the WHO and by three of the four study countries to estimate gestational age, detect fetal anomalies, and identify multiple pregnancies [1]. The first visit thus serves as a unique entry point for screening and early detection of conditions such as hypertension, anemia, infections, and high-risk pregnancies that, if left unidentified, may lead to adverse perinatal outcomes. Despite these recommendations, the quality of care delivered at the first ANC visit remains poorly documented, and few studies have examined whether the completeness of care at this initial contact is associated with perinatal outcomes.* |
